# Supplementary material for: A high Q piezoelectric resonator as a portable VLF transmitter
Source: Nat Commun. 2019 Apr 12;10:1715. doi: 10.1038/s41467-019-09680-2 (PMC6461683; doi:10.1038/s41467-019-09680-2)
Supplement: Supplementary file 1 — Supplementary Information [file 41467_2019_9680_MOESM1_ESM.pdf]

Supplementary Information

A High Q Piezoelectric Resonator as a Portable VLF Transmitter

Kemp, *et al.*

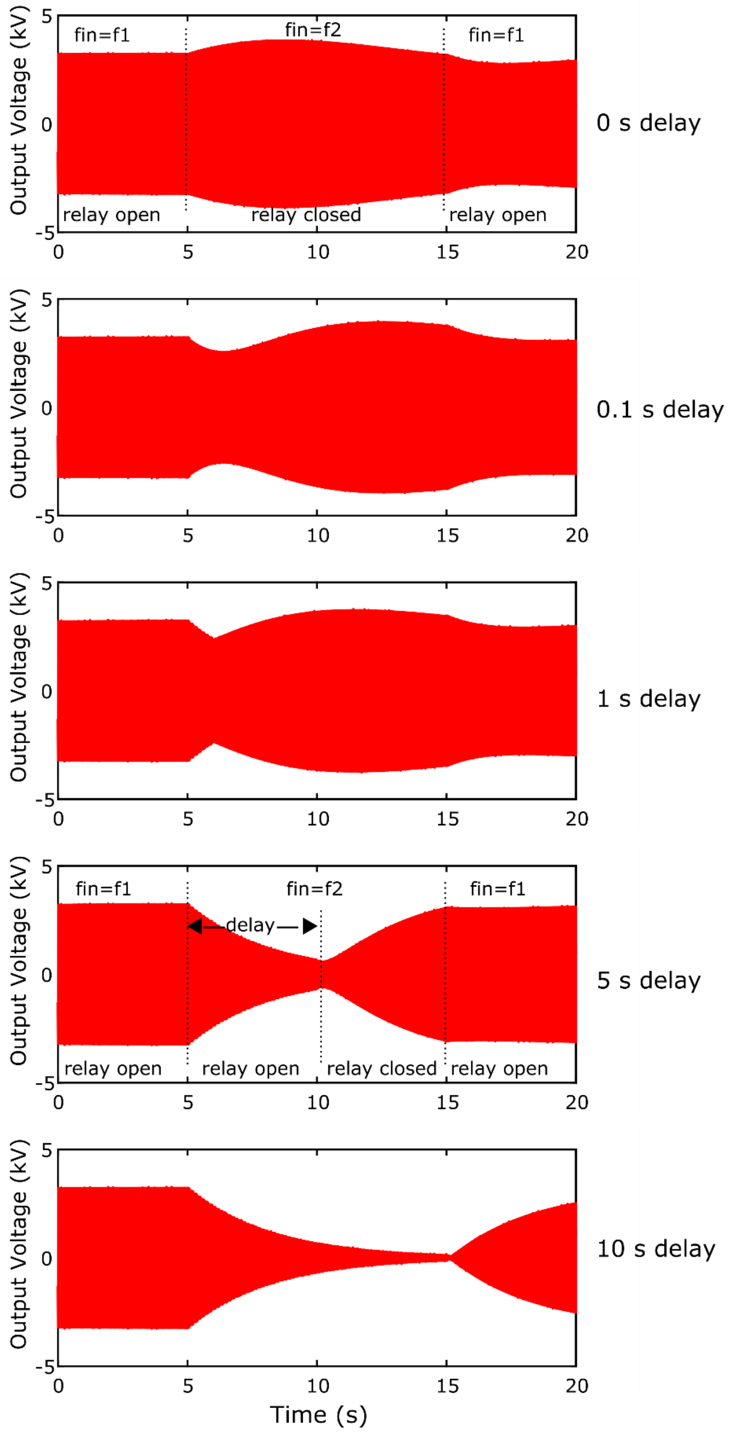

Supplementary Figure 1. Simulated effect of delayed actuation of the modulation relay with a 0.05Hz frequency shift keying (FSK) rate. The 0 s delay case is the same as with direct antenna modulation (DAM), and the 10 s delay case is the same as without DAM. Note that as soon as the relay is actuated, the system begins to ramp up to the new resonant frequency.

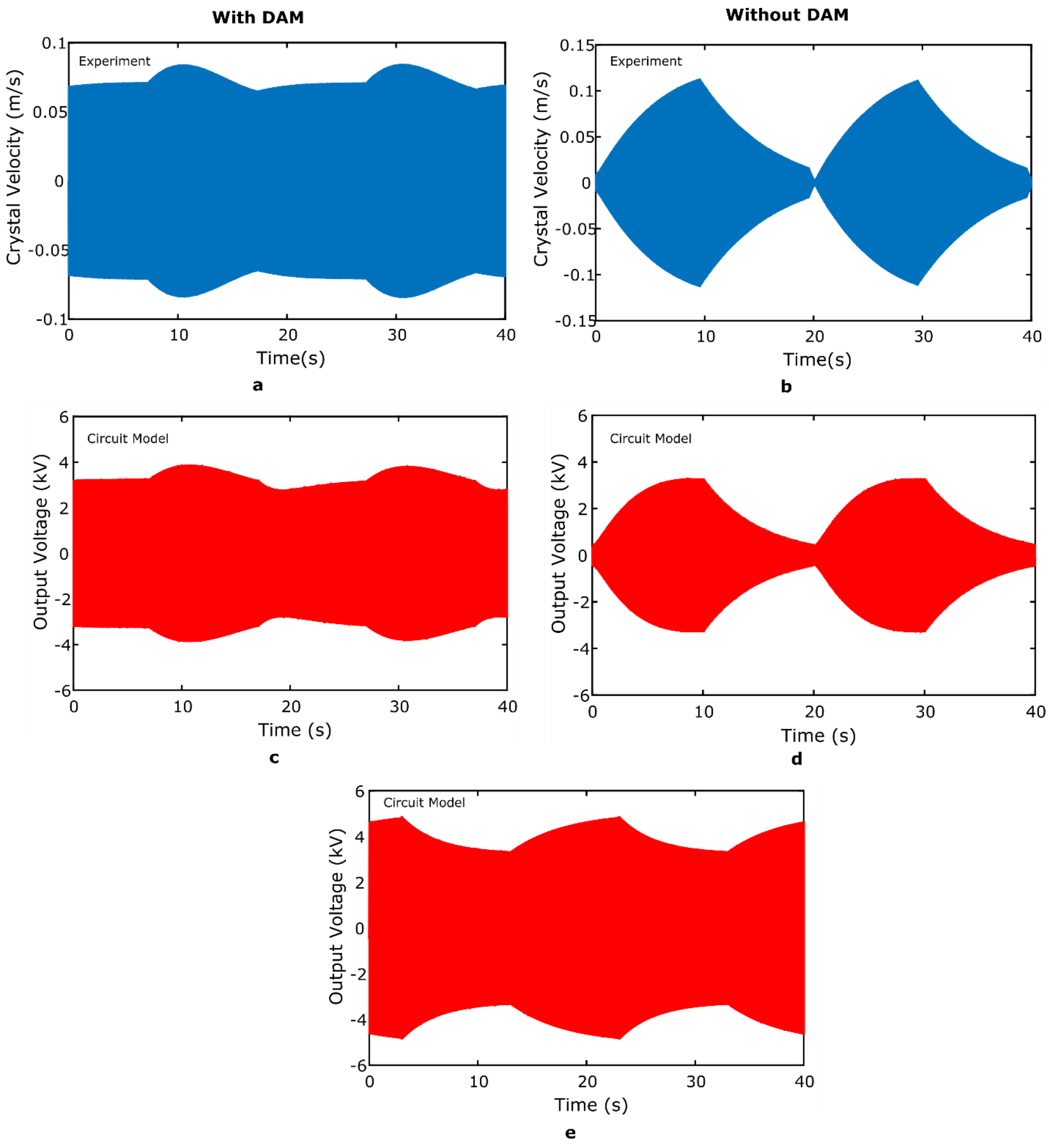

Supplementary Figure 2. Time domain experimental waveforms (a and b) and circuit model simulations (c, d, and e) with (a and c) and without (b and d) direct antenna modulation (DAM). Shown are data with a 0.05 Hz frequency shift keying (FSK) rate. For the experimental waveforms, the output voltage is shown as the response variable while the circuit simulations are the output voltage. In experiment, one of the frequencies was de-tuned slightly to attempt to match the amplitude of the two tones. In (e), the circuit simulation shows the effect if there is no detuning of one of the frequencies.

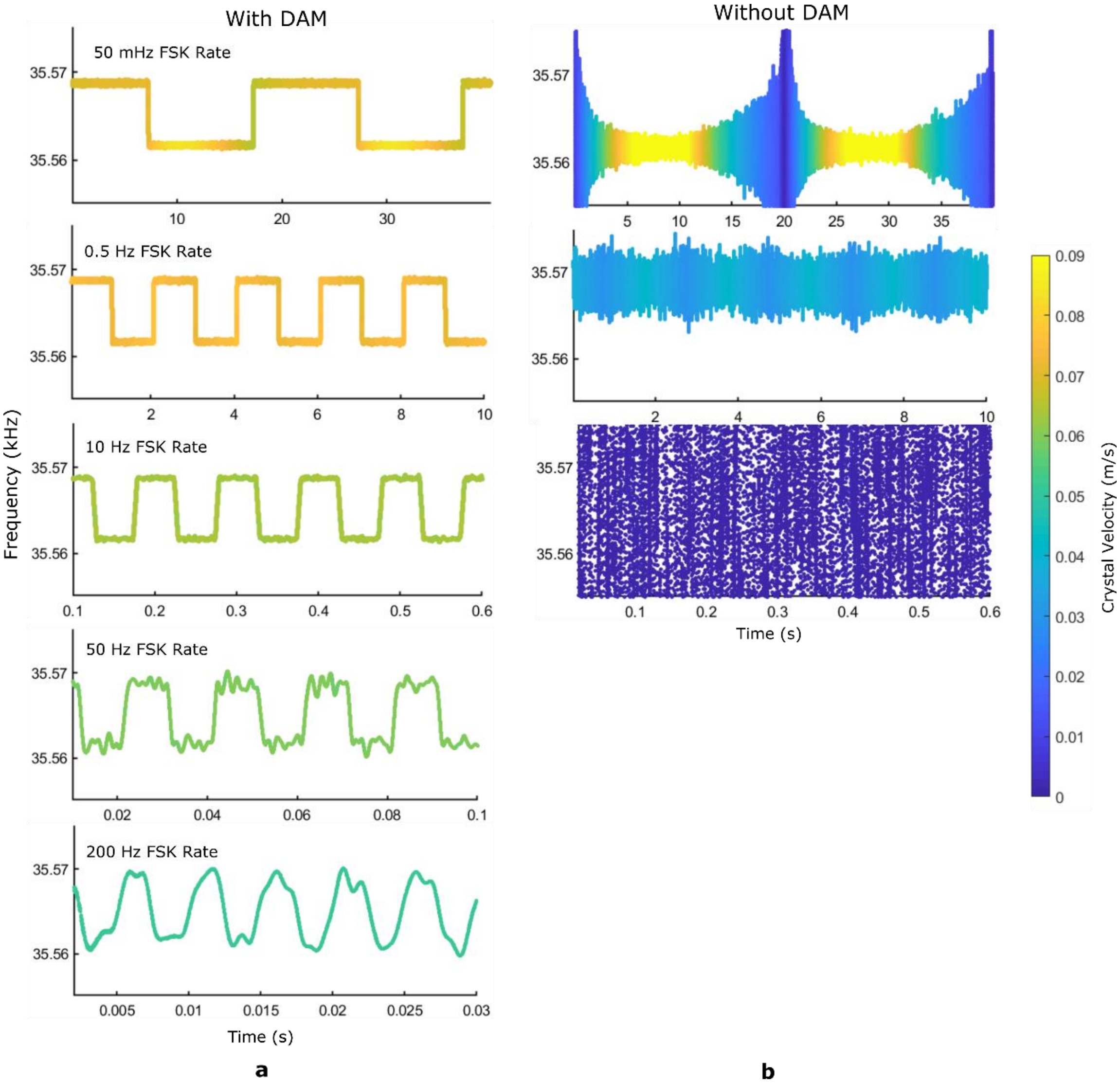

Supplementary Figure 3. Hilbert transforms of crystal velocity (a) with direct antenna modulation (DAM) and (b) without DAM. At the higher FSK rates, the “without DAM” waveforms exhibit minimal delineation of frequency.

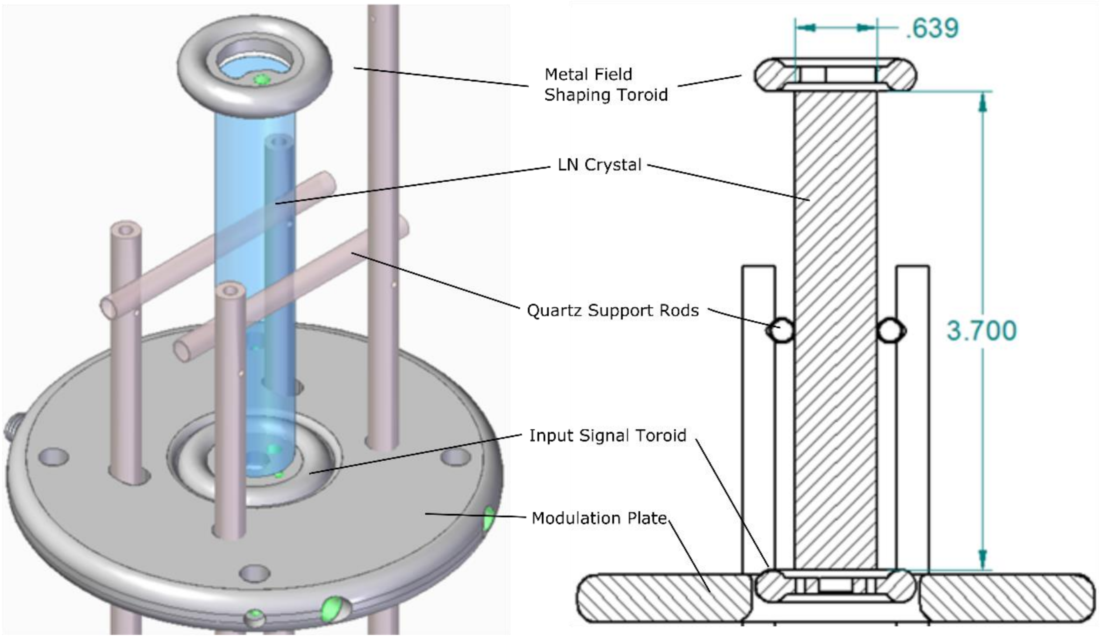

Supplementary Figure 4. Mechanical drawing of mounting, modulation plate, and crystal. Units are inches.

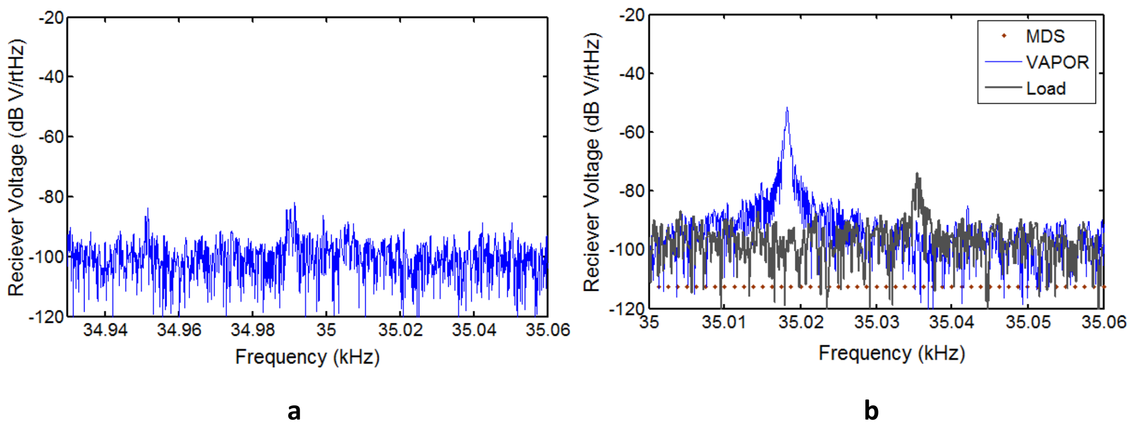

Supplementary Figure 5. Measured noise floor using B field receiver. (a) Background measurement, (b) Measured signal from the piezoelectric transmitter (blue) and a wire-wound resistive load mounted to the battery-powered input signal generator (black). There is >23 dB difference between the transmitter and the RFI from the resistor and input signal generator.

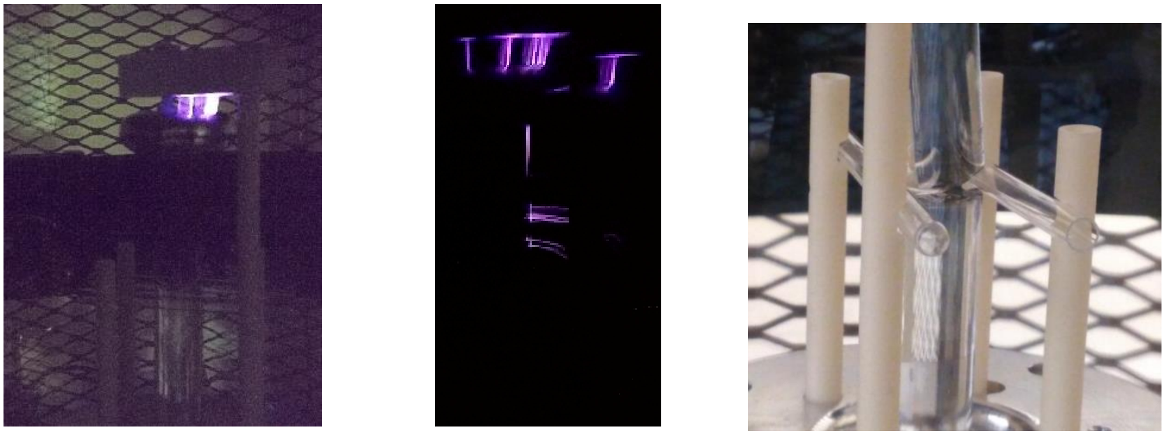

a b c

Supplementary Figure 6. Photographs of the piezoelectric antenna during high-field excitation. (a and b) The air background gas breaks down and discharges form between the corona ring and a mounting post. (c) In an insulating background gas, the crystal fractures after the stress exceeds ~65 MPa.

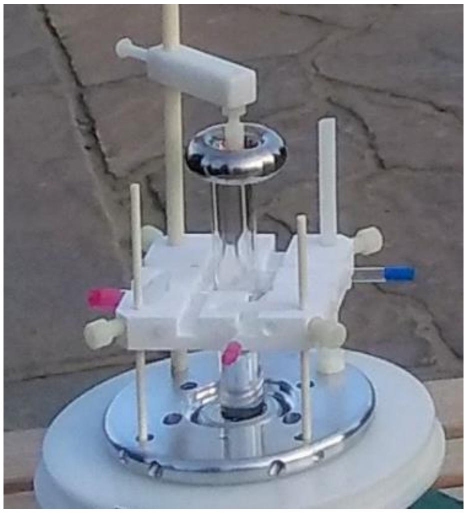

Supplementary Figure 7. Photograph of the prototype piezoelectric antenna during outdoor measurements of the field magnitude versus range.

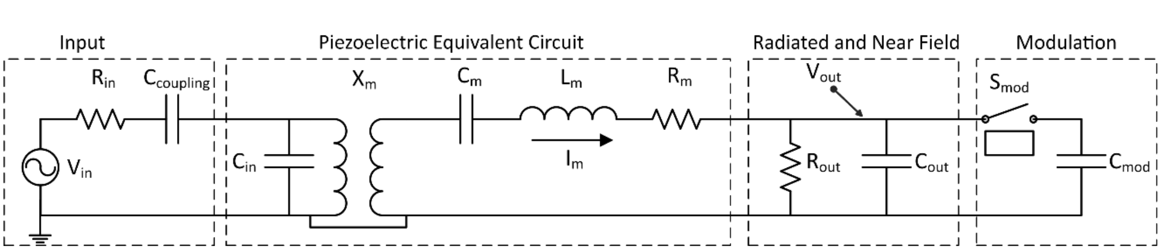

Supplementary Figure 8. Simple circuit model of the piezoelectric resonator system. Shunt coupling capacitance elements omitted for clarity. For the modeling presented in this manuscript, the turn ratio of X_m is 1:1.
